# Optical Waveguide Lightmode Spectroscopy: A Versatile Technique for Real-Time, Label-Free Biosensing

**DOI:** 10.3390/s26041183

**Published:** 2026-02-11

**Authors:** Jeremy J. Ramsden

**Affiliations:** 1 Department of Biomedical Research, Faculty of Medicine and Health Sciences, The University of Buckingham, Buckingham MK18 1EG, UK; j.ramsden@colbas.org; 2 Centre for Molecular Recognition, Collegium Basilea (Institute of Advanced Study), 4053 Basel, Switzerland

**Keywords:** biocompatibility, cytometry, evanescent field, grating coupler, integrated optics, interfacial, lipid bilayers, nonspecific binding

## Abstract

Optical waveguide lightmode spectroscopy (OWLS) is an integrated-optical technique for probing structures at the solid/gas and solid/liquid interface. Spatial resolution perpendicular to the interface is sub-ångström. Thanks to good time resolution, processes involving structural change can also be investigated. This review covers the fundamentals of the technique, the various measurement configurations that are used, interpretation of the primary data received, applications in biosensing, and future prospects.

## 1. Introduction

At the International Symposium on Planning in Science in Prague in 1959, Peter Kapitsa compared artificial measuring instruments (for sound, sight, etc.) with their natural counterparts [1] and drew attention to the fact that for only one sense is the natural sensor better than its artificial counterpart—the sense of smell: think of the nose of the dog, or the tongue of the wine taster. As Scheller and Schubert have pointed out [2], a fundamental problem of analytical chemistry is the detection of a selected substance (the ‘analyte’) at low concentration in the presence of interfering substances. One solution to this problem is to use a high-resolution technique such as high-resolution mass spectrometry (HRMS), in which every substance in the sample is recorded. But the mass/charge ratio recorded for each substance in the sample may not be unique to the analyte of specific interest, and the mass spectrum may turn out to be indecipherably complex, especially if fragmentation of the analyte and interfering substances takes place in the instrument.

Chemical sensors adopt a different approach: to capture, or react with, the analyte and nothing else and transduce the capture event or reaction into an electrical, optical, mechanical or other signal (usually all of these ultimately get converted into an electrical signal). A classic example is the oxide semiconductor gas sensor [3]: interaction of the gaseous analyte with the oxide semiconductor changes the electrical resistance of the semiconductor, from which change the change in ambient gas concentration can be deduced. In order to improve selectivity (i.e., discrimination between the analyte and all other molecules present), as well as enable sensing of larger molecules containing of the order of ten or a hundred atoms, the idea emerged of exploiting Emil Fischer’s ‘lock-and-key’ mechanism [4], either for capture, as in antigen–antibody interaction, giving us an affinity sensor [5], or for reaction, as in a metabolic sensor, in which the biochemical transformation of the analyte yields the sensor signal [2]. Of course, we now know that the ‘lock-and-key’ mechanism is a rather simplistic model of reality [6,7], but at any rate it secured the rôle of biomolecules in chemical sensing.

Whereas the intuitive meaning of ‘biosensor’ is ‘a sensor of a life process’, and this is indeed a definition found in dictionaries (‘a device that monitors and transmits information about life process’ according to Merriam-Webster [8]) and in some of the biosensor literature (e.g., [9]); other dictionaries give a definition restricting the meaning to a chemical sensor that uses biomolecules or, indeed, even a living organism (‘a device which uses a living organism or biological molecules, especially enzymes or antibodies, to detect the presence of chemicals’ in the Oxford English Dictionary [10]). Such devices could, logically, be called biobiosensors.

‘Sensing a life process’ covers a wide range; according to this definition the instruments used in capnography (the real-time measurement of exhaled carbon dioxide), spirometry, pulse oximetry, etc., are all biosensors. ‘Process’ implies temporality, and an appropriate temporal resolution is intrinsic to the value of the measurement. Enzymes have long been used in analytical chemistry (Scheller and Schubert give a good summary of the history of biosensing [2]), but early measurements merely yielded the concentration of the analyte at a particular epoch. The blood glucose sensor based on the Clark oxygen electrode [11] is often considered to be the first biosensor. In this device, the enzyme glucose oxidase reacts specifically with glucose in a blood sample, and the oxygen thereby consumed is monitored with the Clark electrode, from which the glucose concentration can be determined. An important advance was made by Updike and Hicks [12]; they combined the enzyme with the electrode such that the electrons oxidizing the glucose were supplied by an electrical circuit, the current in which yields the glucose concentration [13]. Coupling redox enzymes directly to electrodes became the Holy Grail of early biosensing but turned out to be, in general, elusive [14]. While electrochemical biosensors are still dominant commercially, optical biosensors, which completely avoid the problem of electrochemical coupling, are rapidly growing in importance. In parallel, electrical sensors not based on enzymes but simply on the change in electrical resistance, for example, of a carbon nanotube functionalized to give it selective affinity for glucose, when glucose binds to the nanotube, are being explored. Other examples of affinity sensors in which the result of binding is transduced to an electrical signal include the chemical field-effect transistor (chemFET). Nowadays there is an immense variety of biosensors—sensors for measuring molecules of biological interest [15]. This review focuses on a particular kind of optical biosensor.

The measurement of blood glucose is very important for managing diabetes. In its definition of ‘biosensor’, the Cambridge Dictionary refers to ‘a device that can measure very small amounts of a substance, using chemicals, light, or sound that will react with that substance and send a signal or information to the user, sometimes used in medical devices to measure processes in someone’s body’ [16]. Glucose is, in essence, a biomarker for diabetes, and there is now much activity in detecting diverse biomarkers using CRISPR/Cas technology, which should offer unparalleled specificity [17]. In general, caution is advisable when appraising the possible diagnostic value of biomarkers [18]. Nevertheless, the value of glucose as a biomarker is firmly established, and much effort is now being devoted to wearable biosensing devices for healthcare [19,20]. While battery life is a preoccupation, the ability of the molecular recognition and transducing components of the sensor to perdure is of no less importance, and for this the intrinsic fragility of most biomolecules is a disadvantage. Hence there is also great effort to design and fabricate artificial structures capable of mimicking the exquisite recognition ability of biomolecules [21,22]. For this purpose nanotechnology—engineering with atomic precision—is extremely powerful [23]. Artificial intelligence can be used to design appropriate structures, as well as enhance operational performance [24]. The deployment of nanotechnology makes IUPAC’s insistence that the recognition system of an electrochemical biosensor should utilize a biochemical mechanism [25] seem overly restrictive.

The relationship between the signal output by the sensing device and the concentration of the analyte is somewhat indirect for most current biosensors. This may not be a great disadvantage once a sensor has been fully optimized because one can always construct a calibration curve, although care must be taken to ensure that the composition of the calibration atmospheres or solutions is identical to those anticipated in actual use with samples of unknown composition. With medical biosensors, in many practical cases the overall sample composition is found to remain within fairly narrow limits; hence, sensitivity to non-analyte components may not pose insuperable difficulties since this background remains rather uniform. Evidently the more parameters that one can measure during the sensing operation, the less ambiguous the final determination of the analyte concentration, especially if the sensor output can be directly linked to the analyte concentration via a robust biophysicochemical model. Inevitable trade-offs between sensor complexity—and cost—and informational richness mean that often, especially with devices intended to be deployed in large numbers and disposed of after use, which may comprise only a single measurement, there is a preference for relatively simple devices yielding a single output parameter, which gives little if any mechanistic insight into the analyte binding and signal transduction processes. The efficient development of such ‘minimal sensors’ is greatly facilitated if they can be modeled by a sensor giving more complete information, preferably yielding analyte concentration directly via a biophysicochemical model. Optical waveguide lightmode spectroscopy (OWLS) is capable of providing such information. It is based on a single mode planar optical waveguide, in which one mode of each of two orthogonal polarizations can be excited. Refractive index changes, such as those caused by analyte molecules captured by receptors, on or in the vicinity of the waveguide surface change the effective refractive indices *N* of guided lightmodes propagating along the waveguide. The number of captured molecules can be directly calculated from the two measured effective refractive indices. Furthermore, changes in optical anisotropy—typically originating from changes in orientation—of the captured molecules can also be directly calculated. If living cells are deposited on the optical waveguide, their shapes can be determined, and the kinetics of any shape changes—such as those which may be caused by exposure to a new drug—can be quantitatively determined with excellent time resolution. Since almost any kind of material can be built up on the optical waveguide—only strongly optically absorbing materials, such as metals, are excluded—other kinds of sensors can be modeled and investigated using OWLS and optimized for real-world performance.

Since OWLS is less familiar than electrochemical biosensing, and less familiar than optical biosensors based on light absorption or fluorescence, the purpose of this review is to acquaint researchers wishing to either use a richly informative measurement for investigating life process or those wishing to use a richly informative platform for developing an ultimately simpler biosensor. Section 2 addresses the fundamentals of the technique, Section 3 describes its practical realization, Section 4 examines the problem of design of capture layers, Section 5 briefly outlines some approaches to extracting information from the kinetics of changes, Section 6 deals with measurements of living cells, Section 7 describes further applications of the technique in biosensing, somewhat arbitrarily selected from the vast range of possibilities, Section 8 draws conclusions, and finally Section 9 offers some ideas for future developments.

## 2. Fundamentals of OWLS

**Figure 1:**
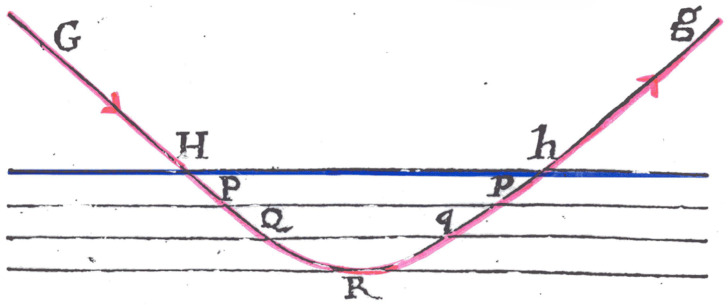
Total internal reflexion, as drawn by Newton [26]. The line Hh (coloured blue by the present author) indicates the glass (above)/air (below) interface. The curved line GHPQRqphg (coloured red by the present author) shows the path of a light ray incident on the interface with an angle exceeding the critical angle.

The fundamental phenomenon is total internal reflexion, already outlined by Newton [26] and verified experimentally [27]: light traveling through glass and reaching a planar interface with air will penetrate a short distance into the air before turning around and re-entering the optically denser medium, provided the angle of incidence (measured from the normal to the interface) exceeds a critical angle (Figure 1).

**Figure 2:**
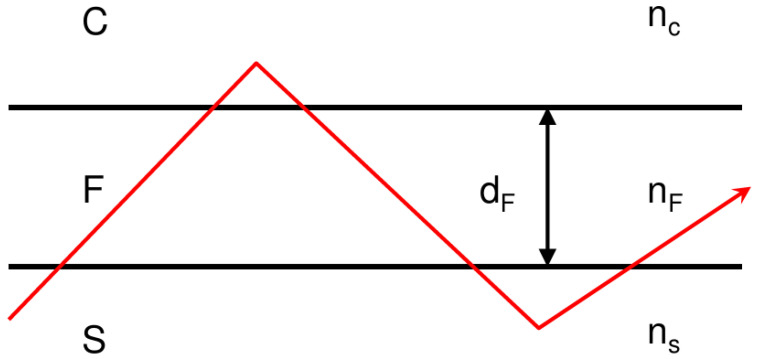
Waveguiding in a slab or channel waveguide of thickness $d_{F}$. The *n* denote the refractive indices of the media, with $n_{F}>n_{S}$ and $n_{F}>n_{C}$, necessary conditions for confining the light in F. The red line represents the propagating guided lightwave as a ray.

**Figure 3:**
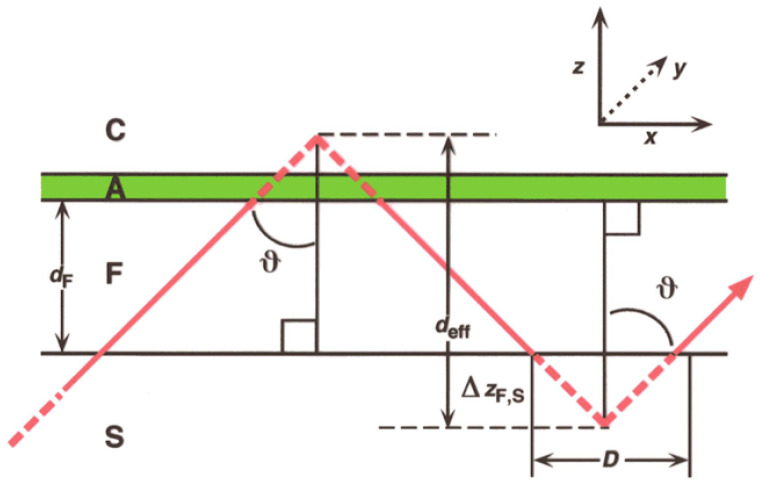
Figure 2 expanded to show an adlayer A (colored green), which may be a capture layer for the analyte of interest. Also shown (inset upper right) is the conventional co-ordinate system used in this paper; the propagation angle θ, which is of course greater than the critical angle for total internal reflexion; the lateral displacement *D* (also known as the Goos–Hänchen shift) upon each reflexion; the penetration depth Δz (shown for the F, S interface) of the evanescent optical field, represented by the pecked portions of the light ray; and the effective waveguiding thickness $d_{\mathrm{eff}}$.

To create a biosensor, the optically dense medium is deposited as a thin film (usually labeled F, typically ∼200 nm thick) onto a substrate (labeled S) of lower refractive index, leaving one surface of the thin film exposed to whatever environment one covers it with (labeled C)—see Figure 2. Very commonly an ultrathin film or adlayer (labeled A, typically only a few nm thick) is interposed between F and C (Figure 3). This adlayer may be composed of receptors for the analyte. In the conventional ray-optic representation of the guided lightwave the total internal reflexion is represented as an abrupt change of direction, whereas in reality the path is presumably curved as in Figure 1. Equivalently, the guided ray can be represented as an electromagnetic field distribution; for the lowest energy (zeroth) mode, the distribution is Gaussian, roughly centered in the middle of the F-layer (exactly centered if the waveguide is perfectly symmetric, i.e., $n_{S}=n_{C}$), with Δz corresponding to the point at which the electromagnetic field intensity has decayed by a factor 1/e from the maximum.

In other words, guided optical waves are arranged to propagate parallel to the interface within the solid phase, and any events taking place within the evanescent field generated by the guided wave at the interface, and decaying exponentially into the cover medium, will change the propagation of the guided waves. ‘Events’ mean reactions, the binding of one molecule to one another already at the interface, adsorption, desorption, etc. Any event that leads to a change in the distribution of electronic polarizability α perpendicular to the interface, i.e., α(z), where *z* is the distance from the interface, is amenable to investigation by this technique. For example, an adsorbing particle, which may be a protein, typically has a polarizability greater than that of the fluid in which it is dissolved or suspended, and thus upon adsorption of such particles the refractive index of the zone in the immediate vicinity of the interface is increased.

**Figure 4:**
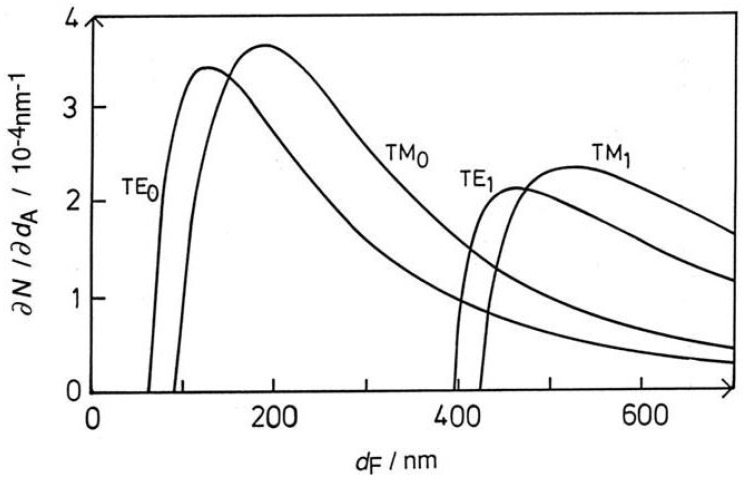
Plots of the sensitivities with respect to adlayer thickness of the effective refractive indices *N* of the zeroth- and first-order transverse electric (TE) and transverse magnetic (TM) lightmodes (wavelength 633 nm) propagating in a planar waveguide of refractive index $n_{F}=1.8$ on a support with refractive index $n_{S}=1.45$ and covered with a medium of refractive index $n_{C}=1.33$, as a function of the thickness of the F-layer. In contrast, if $n_{S}<n_{C}$ (e.g., $n_{S}=1.2$), the sensitivities continue to increase as the waveguide is made thinner, reaching very high values just before cut-off [30].

Waveguiding refers to the confinement of light in a structure that has at least one characteristic length smaller than the wavelength of light. Confinement takes place in a material whose refractive index is higher than that of its surroundings, as in the now ubiquitous optical fibers used for telecommunications. Under such circumstances (cf. the well-known model of a ‘particle in a box’ describing the electron in the hydrogen atom) the light propagates as discrete modes, which are stationary waves within the waveguide, and exponentially decaying evanescent fields beyond it. The characteristic parameter of a guided lightmode is its propagation constant, or, equivalently, its phase velocity or its reciprocal, the effective refractive index *N* pertaining to that mode. The Goos–Hänchen shift *D* and propagation angle θ (Figure 3) are directly related to *N*. Waveguiding is a typical quantum phenomenon, and only discrete modes can exist in a waveguide. For propagation to occur at all, the waveguide must have a minimum thickness, of the order of one third of the wavelength for the lowest energy (zeroth-order) modes. The sensitivity, i.e., the magnitude of the change in propagation constant for a given change in the refractive index profile in the vicinity of the waveguide, increases rapidly to a maximum as the waveguide thickness is increased from the minimum thickness, thereafter slowly falling off [28] (Figure 4). Therefore thin, so-called monomode waveguides are preferred for investigating surface events. The technique is often called optical waveguide lightmode spectrometry (OWLS) since the spectrum of guided modes is measured during the events under investigation, and the energies of the modes are used to calculate characteristic parameters of the event or events taking place within the evanescent field.

Thicker waveguides support higher-order modes and in principle allow the determination of more than two independent parameters in a measurement. For example, if the thickness of a waveguide is increased from about 200 nm, at which the sensitivities of the zeroth-order modes are maximal (Figure 4), to about 500 nm we can then enjoy peak sensitivities of the first-order TE and TM modes, but meanwhile the sensitivities of the TE_0_ and TM_0_ modes fall to about 20% of their peak values, and the peak sensitivities of the TE_1_ and TM_1_ modes are only slightly more than half the peak sensitivities of the TE_0_ and TM_0_ modes [28].

Conventional waveguides (typically made from a thin film of a high refractive index oxide such as niobia or titania, or an organic polymer such as polystyrene, supported on optical silica glass with a refractive index $n_{S}∼1.45$), have $n_{S}>n_{C}$ (both refractive indices being greater than $n_{F}$), and the penetration depth into the cover medium (air or water, with $n_{C}∼1.00$ or 1.33 respectively) is of the order of 100 nm, which is of course more than adequate for probing molecular capture at the F,C (or F,A,C) interface. On the other hand if living human cells are being probed, they are at least a few μm in diameter and may be much larger [29]. For such applications, much greater penetration depths, of hundreds of nm, are useful [30]. They are achievable using so-called ‘reverse symmetry’ waveguides, with $n_{S}<n_{C}$ [31]. For use in aqueous solutions ($n_{C}∼1.33$), this requires a supporting material with an ultralow refractive index; silica aerogels have been found to be convenient and of adequate transparency, and both zeroth- and first-order TE and TM modes have been used to probe living cells with such waveguides [30].

OWLS has obvious similarities with earlier, possibly better known, optical techniques for investigating thin films at interfaces, such as surface plasmon resonance (SPR), ellipsometry and interferometry. These and others are reviewed in [15]; for far-field interferometry see [32]. OWLS enjoys a number of advantages over these other techniques, notably:

1. High sensitivity, i.e., intrinsically at least an order of magnitude higher than that of SPR [33], which is probably the next most sensitive technique. The root of this difference is the short propagation distance, of the order of one micrometer, of the surface plasmons along the thin metal film in which they are generated, whereas the equivalent lightmodes in OWLS can propagate at least three orders of magnitude further in a transparent dielectric. Even higher sensitivities than those attainable with OWLS can be obtained with more recently developed methods such as grating-coupled interferometry (GCI) [34,35,36,37]. Nevertheless, such increased sensitivity is of limited practical value for biosensing since it requires millikelvin temperature control, which is rather problematic. This limitation also applies to interferometry: clearly the sensitivity of an interferometric device can in principle be increased indefinitely by increasing the measurement path length, but practical difficulties soon intervene. Temperature control is discussed further in Section 3.
2. Versatility: It can be used with any transparent solid phase. The waveguide itself must be made from a high refractive index transparent dielectric, but any other medium, such as a lipid membrane, can be coated onto the waveguide as a thin layer. Although, unlike with ellipsometry, OWLS cannot be used with opaque substrata, if, e.g., a metal is required, it can be evaporated as an ultrathin film (of the order of 1 nm thick) onto the waveguide, and the measurement arrangements suitably adjusted to take the attenuation of the guided light into account.
3. Informativity, above all because two independent, orthogonal lightmodes are measured simultaneously. Hence key parameters characterizing the event under investigation, such as the number of adsorbed proteins per unit area, can be calculated directly and straightforwardly from the measured quantities (effective refractive indices) without the need for questionable assumptions or awkward calibration procedures.

To these three one might add the enormous accumulation of knowledge about integrated optics and fiber optics in the context of telecommunications, much of which is applicable to integrated optics for biosensing. The essential difference is that for telecommunications applications, the guided wave is carefully isolated from its environment, whereas in biosensing applications it is deliberately exposed to the environment.

### 2.1. Total Internal Reflexion

Fresnel’s coefficients of reflexion give the ratios of reflected to incident amplitude for beams incident from the high refractive index side (labeled F) onto an interface between two transparent dielectrics, having refractive indices $n_{F}$ and $n_{J}$, with J being either S or C (cf. Figure 2), and $n_{F}>n_{J}$. In general the media may also absorb some light, i.e., the imaginary parts of the refractive indices are nonzero; hence, the refractive indices are actually complex numbers, but typically the imaginary part is very small compared to the real part, and we shall suppose it to vanish. The coefficients are:

(1) $$R_{\rho }=\frac{k_{F}/n_{F}^{2\rho }-k_{J}/n_{J}^{2\rho }}{k_{F}/n_{F}^{2\rho }+k_{J}/n_{J}^{2\rho }}$$

for the perpendicular (s, ρ=0) and parallel (p, ρ=1) polarizations, with

(2) $$k_{F}=kn_{F}\cos\theta$$

where k=2π/λ, the wavenumber for the light *in vacuo* with wavelength λ, θ is the angle of incidence measured from the normal to the interface, and

(3) $$k_{J}=k\sqrt{n_{J}^{2}-n_{F}^{2}\sin^{2}\theta }\;.$$

For a thin film A of thickness $d_{A}$ interposed at the interface (cf. Figure 3), these expressions are modified by summing the reflexions and transmissions at the two interfaces, yielding [38]

(4) $$R_{F,A,C}=\frac{R_{F,A}+R_{A,C}e^{-2i\beta_{A}}}{1+R_{F,A}R_{A,C}e^{-2i\beta_{A}}}$$

where $\beta_{A}$ is the phase thickness of A, defined by

(5) $$\beta_{A}=d_{A}|k_{A}|$$

with $k_{A}$ defined by Equation (3) with J = A. These expressions may be (conceptually) straightforwardly extended to multiple thin layers in an interfacial stack, but before undertaking this rather daunting algebra it should be borne in mind that every new adlayer adds at least two new parameters (thickness and refractive index), and more if the layer is optically anisotropic. These parameters can in principle be measured by using thicker waveguides (cf. the discussion around Figure 4) but, unfortunately, the thicker the waveguide the lower the sensitivities of the measured effective refractive indices to adlayer changes; hence, the potential benefit of measuring higher-order modes needs to be carefully assessed with respect to the problem in hand. Penetration of the light into the optically rarer medium beyond the waveguide is equivalent to the phase shift Φ (Equation (8) below).

### 2.2. The Mode Equations

The relation between the effective refractive indices *N* and the optogeometric parameters of the waveguide can be easily derived from Equations (1)–(3), bearing in mind that *R*, being in general complex, can be written as the product of its modulus and argument:

(6) $$\hat{R}=|\hat{R}|e^{i\Phi }$$

and noting that the existence condition for a guided mode is that the sum of the phase shifts occurring at the two reflexions and upon traversing the width of the waveguide must sum to an integral multiple of 2π; otherwise, destructive interference occurs [28]:

(7) $$\Phi_{S,F}+2\beta_{F}+\Phi_{F,A,C}=2\pi m\;,\;m=0,1,2,\ldots$$

where *m* is the mode number, and the phase changes upon reflectance are derived from Equations (1) and (6):

(8) $$\Phi_{F,S}=-2\arctan\left[{\left(\frac{n_{F}}{n_{S}}\right)}^{\rho }{\left(\frac{N^{2}-n_{S}^{2}}{n_{F}^{2}-N^{2}}\right)}^{1/2}\right]\;,$$

where *N* is the effective refractive index for the considered mode. This equation assumes that the F, S interface is flat and abrupt.

Introducing the normalized propagation constant *b* [39]:

(9) $$b=\left(N^{2}-n_{S}^{2}\right)/\left(n_{F}^{2}-n_{S}^{2}\right)\;,$$

the waveguide asymmetry parameter *a*

(10) $$a=\left(n_{S}^{2}-n_{C}^{2}\right)/\left(n_{F}^{2}-n_{S}^{2}\right)\;,$$

and the dimensionless waveguide parameter *W*:

(11) $$W=kd_{F}\left(n_{F}^{2}-n_{S}^{2}\right)\;,$$

the mode equations for a three layer waveguide (S,F,C) are then

(12) $$\tan\left[W{\left(1-b\right)}^{1/2}\right]={\left(\frac{n_{F}}{n_{S}}\right)}^{2\rho }\frac{{\left(\frac{b}{1-b}\right)}^{1/2}+{\left(\frac{n_{F}}{n_{C}}\right)}^{2\rho }{\left(\frac{b+a}{1-b}\right)}^{1/2}}{1-{\left[b\left(b+a\right)\right]}^{1/2}/{\left(n_{S}n_{C}/n_{F}^{2}\right)}^{2\rho (1-b)}}\;.$$

The lower cut-off (below which no propagation is possible; see Figure 4) is given by

(13) $$W_{c}^{\left(\rho \right)}=\arctan\left[{\left(n_{F}/n_{C}\right)}^{2\rho }a^{1/2}\right]+m\pi$$

and the penetration depth (see Figure 3) by

(14) $$\Delta z=1/{\left[k{\left(N^{2}-n_{C}\right)}^{2}\right]}^{1/2}\;.$$

### 2.3. Useful Solutions

$\Phi_{F,A,C}$ can be calculated *mutatis mutandis* if the adlayer A is assumed to be a uniform, homogeneous film [28]. Since molecular biosensing is mostly concerned with the detection of objects ranging from small metabolites to medium-sized proteins, all of which are much smaller than the wavelength of the visible light typically used in OWLS, the uniform thin-film approximation (UTFA) can be applied to what are actually layers of nanoparticles [40]. In many cases, the adlayer is optically isotropic and characterized by its geometric thickness and a single refractive index $n_{A}$. Measurement of two modes (typically the zeroth-order TM and TE) and the simultaneous solution of the corresponding two mode equations (12) with ρ=0 and 1 enables the thickness and refractive index of the adlayer to be calculated. Explicit solutions for $n_{A}$ and $d_{A}$ are given by Guemouri et al. [41]. For completeness we give them here:

(15) $$n_{A}=n_{F}n_{C}N_{\mathrm{TM}}R^{1/2}$$

and

(16) $$d_{A}=\frac{\left(n_{F}^{2}-n_{C}^{2}\right)\left[\lambda R_{E}-2\pi d_{F}{\left(n_{F}^{2}-N_{\mathrm{TE}}^{2}\right)}^{1/2}\right]}{2\pi \left(n_{A}^{2}-n_{C}^{2}\right){\left(n_{F}^{2}-N_{\mathrm{TE}}^{2}\right)}^{1/2}}$$

where

(17) $$R=\frac{R_{E}-2\pi d_{F}{\left(n_{F}^{2}-N_{\mathrm{TE}}^{2}\right)}^{1/2}/\lambda ]{\left(n_{F}^{2}-N_{\mathrm{TM}}^{2}\right)}^{1/2}}{D_{1}-D_{2}-D_{3}}$$

and

(18) $$D_{1}={\left(n_{F}^{2}-N_{\mathrm{TE}}^{2}\right)}^{1/2}\left(N_{\mathrm{TM}}^{2}n_{F}^{2}+N_{\mathrm{TM}}^{2}n_{C}^{2}-n_{F}^{2}n_{C}^{2}\right)R_{M}\;,$$

(19) $$D_{2}=n_{F}^{2}{\left(n_{F}^{2}-N_{\mathrm{TM}}^{2}\right)}^{1/2}\left(N_{\mathrm{TM}}^{2}-n_{C}^{2}\right)R_{E}\;,$$

(20) $$D_{3}=2\pi d_{F}{\left(n_{F}^{2}-N_{\mathrm{TE}}^{2}\right)}^{1/2}{\left(n_{F}^{2}-N_{\mathrm{TM}}^{2}\right)}^{1/2}N_{\mathrm{TM}}^{2}n_{C}^{2})/\lambda \;,$$

(21) $$R_{E}=\arctan{\left(\frac{N_{\mathrm{TE}}^{2}-n_{S}^{2}}{n_{F}^{2}-N_{\mathrm{TE}}^{2}}\right)}^{1/2}+\arctan{\left(\frac{N_{\mathrm{TE}}^{2}-n_{C}^{2}}{n_{F}^{2}-N_{\mathrm{TE}}^{2}}\right)}^{1/2}$$

and

(22) $$R_{M}=\arctan\left[{\left(\frac{n_{F}}{n_{S}}\right)}^{2}{\left(\frac{N_{\mathrm{TM}}^{2}-n_{S}^{2}}{n_{F}^{2}-N_{\mathrm{TM}}^{2}}\right)}^{1/2}\right]+\arctan\left[{\left(\frac{n_{F}}{n_{C}}\right)}^{2}{\left(\frac{N_{\mathrm{TM}}^{2}-n_{C}^{2}}{n_{F}^{2}-N_{\mathrm{TM}}^{2}}\right)}^{1/2}\right]\;.$$

These two parameters, $d_{A}$ and $n_{A}$, can then be combined to yield the number of particles ν captured per unit area of interface according to

(23) $$\nu =d_{A}\frac{n_{A}-n_{C}}{dn/dc}$$

where dn/dc is the refractive index increment of the particle, dependent on its polarizability and the medium in which it is dissolved or suspended [42].

The two experimentally determined parameters from the zeroth-order TE and TM modes can be interpreted as the ordinary and extraordinary refractive indices of an adlayer, if the thickness is independently determined or deduced from other considerations. Such interpretation allows far-reaching kinetic–structural deductions of the evolution of protein adlayers assembled from solution to be made [43,44].

A more sophisticated approach to evaluating OWLS data makes use of optical invariants. This avoids the results depending in a somewhat ill-defined fashion on the model chosen to describe the adlayer A. For many adlayers encountered in biosensing, especially those formed from macromolecules captured at the solid–liquid interface, the assumption of a uniform, homogeneous film is, actually, not very realistic.

### 2.4. Optical Invariants

The use of optical invariants [45,46,47] enables key parameters characterizing the adlayer to be calculated without the need for possibly unwarranted assumptions. The difference between the optical responses of the idealized Fresnel interface and the real interface is given in terms of surface excess polarization densities [45,47,48] (cf. Gibbs’ surface excess quantities in his treatment of the thermodynamics of thin films). Any measurable quantities must be independent of the position of the (fictitious) Fresnel interface: such optical invariants are obtained by combining the polarizabilities gained by a multipole expansion of the surface excess polarization densities such that the combinations are invariant with respect to displacement of the idealized Fresnel interface. These optical invariants allow determination of the maximum information available from the data with the minimum of ambiguity [47].

Mann [47] has derived the optical invariants for OWLS, given analytic equations relating the parameters obtained from OWLS measurements to the optogeometric parameters of uniaxially anisotropic adlayers and assuming layer homogeneity. The first-order noninvariant excess polarization density parallel to the interface is

(24) $${\tilde{\gamma }}_{e}=kd_{A}{\tilde{n}}^{2}$$

where $\tilde{n}$ is the excess parallel refractive index, i.e.,

(25) $${\tilde{n}}^{2}=n_{∥}^{2}-n_{C}^{2}$$

where $n_{∥}$ is the refractive index parallel to the interface, i.e., the ordinary refractive index, and we can call ${\tilde{n}}^{2}/n_{C}^{2}$ the increment in the relative dielectric constant of the adlayer.

The first- and second-order (in $d_{A}/\lambda$) invariants are:

(26) $$J_{1}=-\frac{N_{\mathrm{FC}}-{\tilde{n}}^{2}-\left(n_{F}^{2}-{\tilde{n}}^{2}\right)\alpha }{n_{C}^{2}/{\tilde{n}}^{2}+1+\alpha }kd_{A}$$

where

(27) $$N_{\mathrm{FC}}=n_{F}^{2}-n_{C}^{2}$$

and the optical anisotropy of the adlayer is

(28) $$\alpha =\frac{n_{∥}^{2}-n_{⊥}^{2}}{{\tilde{n}}^{2}}\;,$$

where $n_{⊥}$ is the refractive index perpendicular to the interface, i.e., the extraordinary refractive index;

(29) $$J_{21}=\frac{n_{C}^{2}}{2}(\frac{{\tilde{n}}^{2}}{N_{\mathrm{FC}}}-1)k^{2}d_{A}^{2}\;,$$

i.e., depending only on polarization density parallel to the interface;

(30) $$J_{22}=-\frac{1-{\tilde{n}}^{2}\left(1+\alpha \right)/N_{\mathrm{FC}}+\alpha /2}{n_{C}^{2}/{\tilde{n}}^{2}+\left(1+\alpha \right)}k^{2}d_{A}^{2}\;;$$

and

(31) $$J_{23}=J_{21}-J_{22}\frac{n_{F}^{2}n_{C}^{2}}{n_{F}^{2}+n_{C}^{2}}\;.$$

The great advantage of OWLS is that four opto-geometric parameters characterizing an (anisotropic) adlayer can be extracted. Table 1 compares the available parameters with those from other optical techniques. Unfortunately a comparable analysis has not yet been carried out for dual polarization interferometry.

If $d_{A}/\lambda$ is small (<0.01), then ${\tilde{\gamma }}_{e}$ and $J_{1}$ dominate the waveguide optical response, and [47]

(32) $$\begin{array}{ccc} {\tilde{\gamma }}_{e} & = & -\frac{N_{\mathrm{FC}}}{N_{\mathrm{FE}}^{1/2}}(\pi m-kd_{F}N_{\mathrm{FE}}^{1/2}\\ & + & 2\arctan{\left(\frac{N_{\mathrm{TE}}^{2}-n_{C}^{2}}{N_{\mathrm{FE}}}\right)}^{1/2}) \end{array}$$

where

(33) $$N_{\mathrm{FE}}=n_{F}^{2}-N_{\mathrm{TE}}^{2}$$

and

(34) $$\begin{array}{ccc} J_{1} & = & \frac{N_{\mathrm{TM}}^{2}-N_{\mathrm{CF}}}{N_{\mathrm{TM}}^{2}N_{\mathrm{CF}}/n_{F}^{2}}\{{\tilde{\gamma }}_{e}-\frac{N_{\mathrm{FC}}}{N_{\mathrm{FM}}^{1/2}}[\pi m-kd_{F}N_{\mathrm{FM}}^{1/2}\\ & + & 2\arctan(\frac{n_{F}^{2}}{n_{C}^{2}}{\left(\frac{N_{\mathrm{TM}}^{2}-n_{C}^{2}}{N_{\mathrm{FM}}}\right)}^{1/2})]\}\;, \end{array}$$

where

(35) $$N_{\mathrm{CF}}=\frac{n_{F}^{2}n_{C}^{2}}{n_{F}^{2}+n_{C}^{2}}$$

and

(36) $$N_{\mathrm{FM}}=n_{F}^{2}-N_{\mathrm{TM}}^{2}\;.$$

The adlayer mass *M* per unit area, for thin films, i.e., $d_{A}/\lambda ≲0.01$, is given by

(37) $$M\approx -{\tilde{\gamma }}_{e}\left(dc/dn\right)/k\;.$$

It may often be the case that a thin film formed by adsorbing proteins is of unknown structure. It cannot be assumed that it is isotropic, but judicious use of a quasi-isotropic approximation may allow particles to be counted with acceptable error [49].

## 3. Practical Measurement Arrangements

In this section we deal exclusively with grating incoupling since this is overwhelmingly the most popular realization of OWLS. Other schemes are covered in [50]; far-field interferometry is covered in [32]. An optical grating is incorporated into the waveguide at the F, S or F, C interface. A shallow grating, a few nm deep, is sufficient for coupling the few percent of light needed for measurement purposes (i.e., high coupling efficiency is not necessary—cf. [51]), and it is convenient if, for hydrodynamic purposes, the surface remains essentially planar, as it does for a grating depth of a few nm and a grating constant of a few hundred nm. Perfect polarity can be achieved if the grating is fabricated by refractive index variation (e.g., by implanting impurity ions with the appropriate spacial modulation) rather than by changing the topography [52].

**Figure 5:**
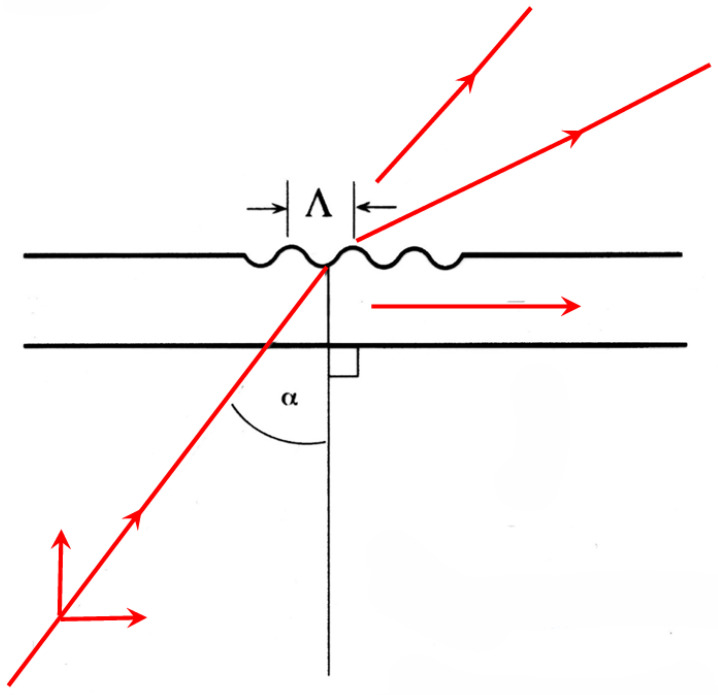
OWLS incoupling into a planar waveguide (cf. Figure 2) using a grating with constant Λ. The red lines show the incoming external beam incident with angle α onto the normal of the grating and the spacial harmonics generated by the grating.

In a typical incoupling configuration (Figure 5), an external beam impinges onto the grating, making an angle α with the grating normal. The wavenumber component in the direction of guided propagation is then $n_{\mathrm{air}}\sin\alpha +2\pi \ell /\Lambda$, where ℓ=0,±1,±2,… is the diffraction order and Λ the grating constant. If this matches a guided mode with wavenumber kN, incoupling will occur according to the coupling condition

(38) $$N=n_{\mathrm{air}}\sin\alpha +\lambda \ell /\Lambda \;.$$

**Figure 6:**
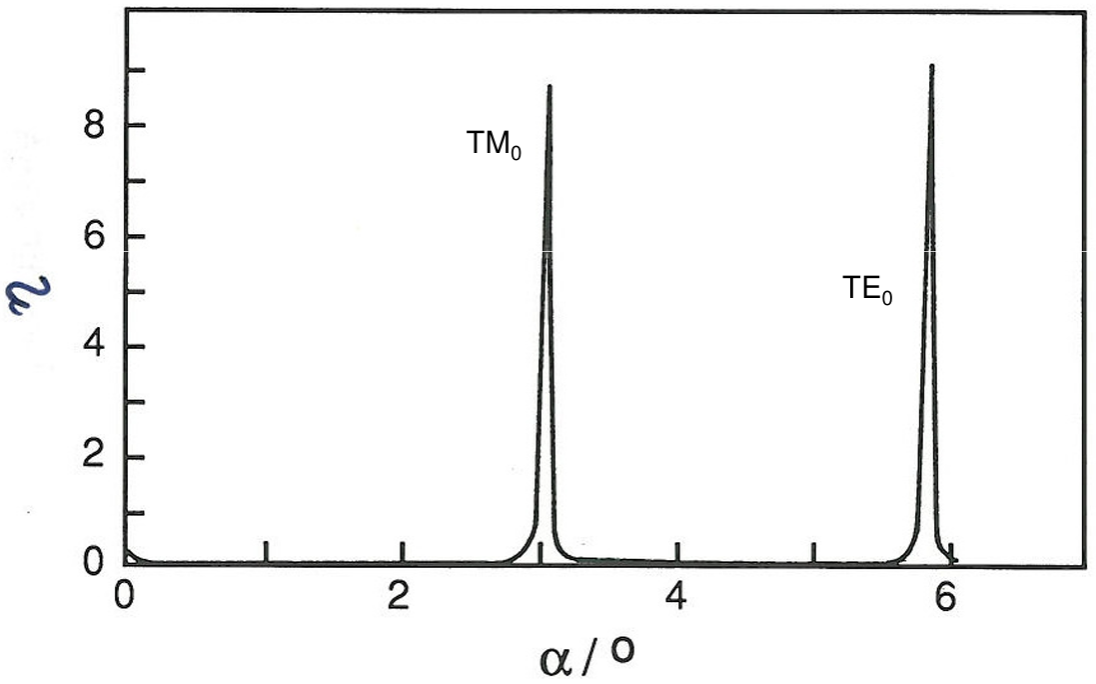
Typical OWLS mode spectrum, showing incoupling efficiency η versus incoming angle α.

The measurement procedure is therefore to record light emerging from the end of the waveguide while varying α. The emerging light will appear as a series of sharp peaks, successively TM_m=0,*ℓ*=1_, TE_m=0,*ℓ*=1_, etc. (Figure 6). At the time of writing, the highest precision in the determination of *N* is achievable by mechanical goniometry in a temperature-controlled environment $\Delta T_{\mathrm{air}}=\pm 1$ °C, hence $\Delta n_{\mathrm{air}}=\pm 10^{-6}$), with which α can be determined to microradian precision. A very monochromatic light source is required—see Table 2.

The development of low-cost embossed precision grating couplers of a standard composition was a major breakthrough in the development of OWLS [53,54]. Prior to that, the production of gratings was a very laborious and expensive process [55]. Roll-to-roll technologies with organic polymeric waveguides potentially offer even lower cost [56]. Very often a variety of substrates is required for biosensing purposes. It was found that depositing a film 10 nm thick on a standard, mass-produced waveguide was adequate for mimicking the bulk [57] and hence provides a strategy for conveniently fabricating a great variety of different surfaces for biosensing.

### Effect of Temperature on Waveguide Parameters

The refractive index of most substances varies significantly with temperature. The variation is directly related to the coefficient of thermal expansion of the substance, as can be seen from the Lorentz–Lorenz equation, which connects the macroscopic index of refraction with various atomic or molecular parameters, i.e., molecular mass $M_{r}$, density ρ and molar refractivity $R_{M}$:

(39) $$n^{2}=\frac{M_{r}+2\rho R_{M}}{M_{r}-\rho R_{M}}\;.$$

Of these various terms, only the density varies with temperature. It follows that the temperature in any biosensing procedure should be kept as uniform as possible [58]. Among the various materials associated with the procedure, water (from which the cover medium is usually constituted) has the greatest coefficient of expansion. Since $\partial N/\partial n_{C}∼0.1$ and $\partial n_{C}/\partial T∼10^{-4}$ K^−1^ for water, aqueous solutions should be thermostated to ±0.1 °C to keep them within the bounds of uncertainty imposed by other factors involved in the determination of the lightmode spectrum peaks (see Table 2). After applying the usual combining laws it can be seen that all factors contribute roughly equally to the overall uncertainty in *N*, typically ±1–2 $\times 10^{-6}$.

**Table 2:** Contributions to the uncertainty of *N* in an input grating coupler.

Parameter	Typical Value	Uncertainty	Physical Origin
$n_{\mathrm{air}}$	1.0002673	$10^{-7}$	Temperature fluctuations (±1 °C)
α	0.09 rad	$1.25\times 10^{-6}$ rad	Goniometer (mechanical instability)
λ	632.816 nm	0.001 nm	Laser mode jumping
Λ	416.147 nm	0.001	Grating lateral thermal movement

Temperature, of course, affects not only the waveguide parameters but also the thermodynamics and kinetics of the binding processes involved in biosensing and the characteristics of transport of analyte to the sensor surface due to the temperature-dependent viscosity of most solvents, especially water, which makes it even more important to ensure that the biosensing operation is carried out at a stable temperature without gradients.

## 4. The Design of Capture Layers

We have seen that the prototypical biosensor comprises a film, typically thin, for abstracting the analyte of interest from potentially a complex sample, and a transducer for converting the number of captured analyte molecules into one or more physical measuranda [59] (the effective refractive indices *N* in OWLS). The classic thin-film-based biosensor comprises simply an array of receptor molecules (such as antibodies, if it is desired to measure an antigen, or an array of antigens if it is desired to measure an antibody) coating the transducer, which might be a microcantilever in the case of a mechanical transducer, carbon nanotubes in the case of an electrical resistive transducer, the electrode coating a piezoelectric crystal in an acoustic transducer (such as the quartz crystal microbalance), or a planar optical waveguide in the case of OWLS. Associated with this design is an extensive chemistry of protein immobilization [60]. Whereas for scanning probe microscopy the main immobilization requirement is simply to prevent the protein under investigation from being displaced by the scanning tip [61], for biosensing the most important aspect is that the site of the immobilized molecule that functions as the receptor for the analyte remains as close to its native conformation as is possible and, furthermore, oriented in such a way as to enable unhindered access by the analyte. These requirements may be quite difficult to meet. Similar considerations apply, *mutatis mutandis*, to other kinds of receptors suitable for use in biosensors such as aptamers—small single-stranded nucleic acid (usually RNA) polymers that are able to adopt configurations binding a target analyte with high affinity, which can be optimized using evolutionary screening (well established as the ‘systematic evolution of ligands by exponential enrichment’, SELEX, process) [62].

For many years, nonspecific binding in antigen–antibody assays—where the analyte binds other than to the receptor, or when molecules from the sample matrix bind to the transducer surface—was considered to pose a great problem, and many strategies were adopted in an attempt to diminish the problem. One strategy is to prepurify the sample prior to exposing the biosensor to it [63], but this rather defeats the point of using the biosensor. Another strategy is to lower the nonspecific affinity of the transducer surface [64]. While it has had some successes, as Breault-Turcot et al. have remarked, ‘the mechanism for the reduction [in nonspecific adsorption] is still debated’ [65]. Yet another strategy is to expose the biosensor to a so-called ‘blocking agents’ (serum albumin has been very commonly used) prior to applying the sample, the idea here being that the blocking molecule binds to everything except the specific analyte receptor and, once bound, exhibits a low affinity for everything else. Introducing significant quantities of a new component to the system under investigation tends to make it harder to interpret the results of the biosensing measurement. The most sophisticated attempt to overcome the apparent problem of nonspecific adsorption has been an ingenious and elaborate extension to OWLS, namely, focal molography (the name is a contraction of ‘molecular holography’), in which an ordered array of receptor molecules is created on the waveguide surface such that light is effectively focused in a certain way that is changed when analyte binds to the receptors [66,67,68].

All of this appears, however, to be based on a misconception rooted in faulty design of the receptor layer. If the receptor is allowed to saturate the transducer surface—for random sequential adsorption [69] this would correspond to the jamming limit—there is no further space on the surface where anything could bind. Hence, with a properly designed receptor layer, there is no nonspecific binding. This was strikingly illustrated in a seminal experiment [70], the primary purpose of which was to devise a biosensor for detecting promastigate surface protease (PSP) antibodies. PSP is abundantly expressed on the surface of the protozoan parasite *Leishmania major*; hence, an infected subject has antibodies to PSP circulating in their blood, and they will be captured by a receptor layer of PSP (immobilized in a lipid bilayer) coating an optical waveguide, continual measurement of the effective refractive indices of two modes of which enables the number of bound antibodies, hence their concentration in the sample, to be evaluated at any epoch. The results of the experiment are summarized in Table 3. They are highly revealing. The PSP itself adsorbed to reach a definite monolayer. The importance of the membrane anchor was shown by the almost complete lack of adsorption of a modified form of PSP from which the anchor had been enzymatically removed. Anti-PSP antibodies bound very strongly to the PSP layer, whereas a randomly selected antibody practically did not bind at all. On the other hand, antibodies did bind to a membrane not coated with PSP (so-called ‘nonspecific’ binding).

Two important conclusions from this work are: (i) if receptors adequately cover the surface, there is no need to add a so-called ‘blocking’ agent (serum albumin is often used for this purpose) to prevent nonspecific binding, even if the uncovered surface as a high affinity to the analyte, because the surface is inaccessible to the antibodies after being coated with the antigen; (ii) precise quantification using OWLS of every step in the assembly of the biosensor was indispensable for appropriately designing the fabrication procedure. Such an approach is applicable not only to biosensors based on OWLS, but also to the optimization of capture layers and appropriate hydrodynamic sensing regimens for practically any kind of biosensor, whether based on electrical, optical or mechanical transduction, provided that the surface of the biosensor can be mimicked on the surface of the optical waveguide.

Nonspecific binding, if it does occur (for example, if the waveguide is incompletely covered with analyte receptors), is somewhat analogous to fog in silver halide emulsion-based photography [71], and a similar strategy to depress it can be adopted in biosensing, namely, to introduce a minimum threshold for binding to occur [72]. A straightforward way of achieving this is to select an analyte that is a multidentate ligand for the receptor constituting the capture layer. An IgG antibody is bidentate, and conditions of binding to a layer of antigen can be chosen such that an antibody binding to only one antigen will remain bound very transiently, whereas if the IgG binds to two antigens it will remain bound indefinitely. If nonspecific binding is fully reversible, it has no effect on the detection efficiency of the biosensor [72].

Another strategy for capture layers has been to create a rather thick (∼100 nm) layer of a hydrogel such as carboxydextran on the surface of the waveguide and covalently link the receptor proteins to the hydrogel. This has been especially popular with surface plasmon resonance biosensors. Possibly it was originally motivated by the notion that one could enhance the effective sensitivity because analyte capture took place within nearly all of the evanescent field rather than just at a thin layer immediately adjacent to the waveguide surface (albeit where the evanescent field is most intense). Unfortunately the transport of molecules through a hydrogel is exceedingly complex [73], which makes it very difficult to interpret measured kinetics of binding and release (see Section 5). Furthermore, carboxydextran is a polyelectrolyte with Lewis basic character and hence profoundly affects the structure of the water in which the analyte binding events take place, which in turn will inevitably affect the thermodynamics and kinetics of the binding events. It becomes, therefore, difficult to relate the association and dissociation rate coefficients derived from the polyelectrolyte-coated biosensor to those from measurements under better-defined conditions, such as a regime of convective diffusion [74], or in homogeneous solution.

Cell membranes are usually richly decorated with a great variety of proteins, which are typically anchored by a stretch of polyamino acid adapted to strongly embed itself in the lipid bilayer. An example is promastigate surface protease (PSP), discussed above. This suggests that an attractive way to immobilize proteins while maintaining their native state is to anchor them to lipid bilayers [75]. Many proteins are found naturally bound to lipid bilayer membranes by means of a suitable oligopeptide or other kind of anchor, and for those proteins that do not have it, modifying them to provide an anchor may feasibly involve a straightforward genetic engineering procedure.

## 5. Interpretation of the Kinetics of the Sensor Output

Because of their ability to deliver exquisitely high-resolution kinetic data, biosensors are indispensable for gaining deeper mechanistic insight into biochemical binding than is possible from the simple determination of an affinity constant. Different values of binding and release constants have very different mechanistic connotations even if they have the same ratio.

For most biosensing applications it is customary to arrange a cuvette above the grating such that it forms the floor of the cuvette. Knowledge of the precise geometry of the cuvette, and precisely controlled and appropriate (laminar) flow rates of liquids or gases, establishes a convective diffusion regime [74], in which the rate of arrival of analyte molecules at the receptor adlayer (cf. Figure 3) is well defined. The kinetics of accumulation of analyte (i.e., a graph of ν(t)) then yield the sought-for analyte concentration in the sample [76,77]. This approach has been extensively used to characterize proteins in solution. Analyte capture may then be followed by a purge step, in which analyte-free medium flows above the grating, from which information about the reversibility of receptor–analyte interaction may be obtained.

Orgovan et al. have written an excellent and comprehensive review of sample fluidic handling [78]. Naturally there are many subtleties involved in the kinetic analysis of judiciously planned series of experiments. For example, different sample dilutions may be required to confirm an interpretation of results; different ionic strengths, achieved with different ions, may help to unravel complex interfacial forces. Saftics et al. have written an excellent and comprehensive review of data evaluation methods [79].

Since the effective refractive indices are determined by mechanically scanning incoupling angles, it may take tens of seconds to scan both TE and TM modes. With mechanical scanning there is a trade-off between speed and accuracy; hence, it may be inadvisable to scan faster. On the other hand very fast but small changes in effective refractive index can be monitored by positioning the incoming beam on the flanks, where α varies almost linearly with *N* (cf. Figure 6). The time resolution of the measurement now depends only on the electronic circuitry (analogue–digital conversion).

## 6. Cytometry

It is a natural extension of the capture of molecules, including proteins, at the waveguide surface to capture living cells. For example, a biosensor for infectious diseases [80] could be created by coating an optical waveguide with receptors specific for selected pathogenic bacteria (e.g., suitably immobilized bacteriophage), exposing the coated waveguide to a biofluid of the patient, and counting the number of captured bacteria [81]. The concept can readily be extended to eukaryotic cells, for example, in cancer detection [82,83].

Early attempts to use living cells in biosensors were indirect—the cells carried out a transformation of the analyte, and the products were measured using conventional means [84]. Far more sophisticated is the direct interfacing of the cell to the optical waveguide [85]. The effective refractive indices are exquisitely sensitive to the shape of the cell reposing on the waveguide. Since the optical evanescent field decays exponentially, the effect of shape is determined by the Laplace transform of the cross-section of the cell A(z) taken parallel to the plane of the optical waveguide [86], this being the effective volume $v^{\prime }$ of the cells; i.e.,

(40) $$v^{\prime }=\int_{0}^{\infty }A\left(z\right)\exp\left(-z/\Delta z\right)ẓ$$

where 1/Δz is the decay constant of the evanescent field given in Equation (14). If cell volume is preserved, as it must be over short intervals, a single parameter suffices to characterize the cell, dimensions such as contact area and diameter all having a fixed relation to each other. It must, however, be emphasized that this interpretation rests on strong assumptions not only of stable cell volume but also of unchanging details of refractive index distribution within the cell and its adhesion geometry. Whether the likely violation of these assumptions in a real biological system vitiates the interpretation needs to be carefully examined for each system investigated. If cell shape is unchanging over the duration of the experiment, other phenomena may contribute to changes in the effective refractive indices of guided lightmodes, such as changes in internal refractive index distribution (e.g., due to cytoskeletal rearrangement).

A very characteristic response of cells to surfaces is the shape transition from spheroidal (when freely suspended in solution) to segmental (‘spread’). The high-resolution determination of spreading kinetics provides an extremely useful characterization of cell state [87]; if phase plots of two parameters extracted from the OWLS measurements are constructed, behavioral differences between different cell states (e.g., different states of differentiation) are immediately apparent. Adhesion kinetics can similarly be used to classify cells [88]. OWLS has also been used to determine metabolic state [89].

When experimenting with cells, it is often convenient to carry out massively parallel experiments, and this can be accomplished using multiwell OWLS [82]. Standard 384-well format cell assay microplates are now available in which each individual well is equipped with a 2×2 mm grating. These gratings are simultaneously illuminated from below the well at a fixed angle of incidence using either a broadband source or rapidly sweeping the wavelength α of a diode laser (typically over a range of 15 nm). At a certain wavelength $\lambda_{r}$ light is incoupled; this wavelength depends on the effective refractive index (cf. Equation (38)—it is just rearranged) according to

(41) $$\lambda_{r}=\Lambda \left(N-n_{C}\sin\alpha \right)/\ell$$

and this incoupled or resonant light (hence the arrangement is sometimes called a resonant waveguide grating, RWG [90,91]) is outcoupled by the same grating and detected by a CMOS or CCD camera [92]; for massive parallelization it is more convenient to sweep wavelength than angle, and the swept range is short enough for the assumption of constant refractive indices of system components to be reasonable. It should of course be checked that there is no strong absorption peak of any component in the range. A commercial system, the Corning Epic BT, uses a wavelength range centered on 828 nm and the zeroth-order TM mode is excited. Since only one lightmode is measured, one sacrifices information compared with classic OWLS but, as already pointed out, under conditions of constant cell volume a single parameter suffices to characterize shape. In some cases a rigorous interpretation of the measured $\lambda_{r}$ has not been undertaken, but instead the resonant wavelength shift Δλ occurring when some change in the system takes place has been interpreted empirically with the help of parallel classical OWLS experiments [93], and this suffices for many purposes such as large-scale screening of potentially therapeutic compounds to hinder cancer cell adhesion. Even simpler operation is possible by choosing a wavelength near the cut-off [94]; one then merely needs to measure the presence or absence of outcoupled light.

## 7. Further Practical Applications

This section complements the applications that have already been mentioned illustratively in the preceding exposition.

### 7.1. Three-Layer Waveguides

In this configuration (Figure 2), the grating coupler acts as a refractometer of the ambient environment. The mode equations (12) (with ρ=0 or 1) for a three-layer waveguide are solved for $n_{C}$; a gaseous or liquid environment is normally isotropic; hence, measurement of a single mode suffices. The result is then interpreted with the help of the Lorentz–Lorenz Equation (39).

Typically a series of experiments would begin with finding the thickness and refractive index of the (normally amorphous and isotropic) F-layer. If the support is made from a standardized material with an accurately known refractive index, there is no need to determine $n_{S}$ experimentally. The determination of two effective refractive indices in the presence of a known inert atmosphere (e.g., dry nitrogen at room temperature, or air with known temperature and humidity) enables $n_{F}$ and $d_{F}$ to be found by simultaneously solving the two mode equations corresponding to the TE and TM modes.

Depending on the relative temperatures of atmosphere and waveguide, vapors present in the waveguide may condense on the surface of the F-layer to form a thin film of condensate [95]. It can be quantified by simultaneously solving the two four-layer mode equations corresponding to the TE and TM modes. Selectivity can be achieved by tuning the temperature of the waveguide; by applying a gradually decreasing temperature gradient, the spectrum of vapors present in the ambient atmosphere can be obtained.

Water condensing from exhaled humidity has been used to monitor breathing [96]. For medical investigations, it may be very useful to use a universal technique like OWLS rather than a specialized humidity monitor [97] since appropriately sensitized waveguides can be used to selectively detect a practically unlimited range of exhaled metabolites.

The waveguiding films are typically made by the pyrolyzed sol–gel process [54] or by sputtering [98], which results in a nanoporous material capable of accepting guest molecules. Comparison of $n_{F}$ in the presence of either water or heavy water enabled the porosity to be determined [99]—around 15% seems to be typical. This approach can be used for material investigations, for example, to investigate the interaction of the F-layer with small guest molecules. Many different materials can be investigated—the only requirements are that they are transparent at the wavelength of the guided modes and that their refractive index exceeds that of the support layer S. The sol–gel process typically requires considerable empirical optimization in order to create good-quality films; hence, there is strong motivation to make the grating couplers from only one kind of material, and physical vapor deposition may be more convenient if a great variety of materials is to be studied.

If it is important that the F-layer is unresponsive to its environment, it may be practical to evaporate a dense thin film of another material on the surface of the F-layer [57] in order to prevent access to the nanopores from the cover medium. This strategy is also useful if it is necessary for the surface in contact with the environment to have particular chemical characteristics (the sole desired physical characteristic of the surface is for its roughness to be as low as possible). While the resulting structure is technically a four-layer waveguide, the adlayer would normally be unchanging during subsequent biosensing, and its (fixed) parameters can be determined at the start and typically subsumed into those of the F-layer.

### 7.2. Four-Layer Waveguides—Uniform Layers

**Figure 7:**
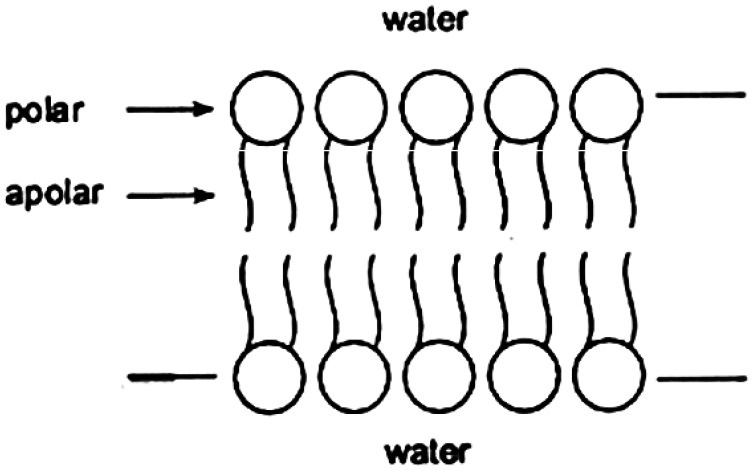
A bilayer lipid membrane formed from amphiphilic molecules such as the phosphatidylcholines. When deposited on a hydrophilic metal oxide waveguide, strong interactions between water and the polar head groups (drawn as circles) of the lipid molecules (their alkyl groups are drawn as wavy lines) and the hydroxyl groups covering the waveguide surface ensure that the lower region of water, although thin, maintains a biomimetic environment for the membrane.

The bilayer lipid membrane (Figure 7) is indubitably a biological structure and very attractive as a receptor for biosensing because of its tremendous versatility. Natural lipids can be complemented by synthetic ones, and then there is the enormous variety of prokaryotic lipids to choose from. The Langmuir–Blodgett technique is convenient for applying a lipid bilayer to the waveguide surface. Alternatively, lipid bilayers can be formed in situ by allowing lipid vesicles to collide with the solid–liquid interface [100]. Membranes can be formed from a single type of phospholipid, or from mixtures, and may include other types of lipids such as cholesterol. Because of their amphiphilic structure, lipid bilayers can function as receptors for amphiphilic molecules, such as many drugs; hence, a lipid-coated grating coupler can function as a biosensor for such drugs [101,102]. Partitioning of the drug into the lipid changes its refractive index and, hence, the effective refractive indices of the lightmodes.

### 7.3. Further Examples of Discrete Receptors

The immobilization of protein receptors on the waveguide surface using a planar lipid bilayer is just one example of innumerable technologies to affix proteins (typically antigens or antibodies) or other kinds of biopolymers such as nucleic acids, including aptamers, and polysaccharides to the waveguide surface [103]. As already discussed in Section 4, the lipid membrane is highly attractive because it provides a natural anchoring medium, avoiding denaturation, which is rather likely to occur if the protein is simply allowed to adsorb on the bare waveguide surface [104], and misorientation (such that the binding site for the analyte becomes inaccessible), both of which vitiate the capture function of the receptor layer. The ultimate choice of immobilization strategy depends on the details of the biosensing problem being addressed. Even difficult matrices such as food can be addressed [105,106].

DNA and RNA can be readily immobilized on waveguide surfaces, opening the way to an enormous field of investigation of transcriptional control, based on the high-resolution monitoring of the association and dissociation of transcriptional activators to and from the nucleic acid, possibly in the presence of antibiotics, and their influence on the subsequent binding of RNA polymerase [107].

### 7.4. Metabolic Sensors

OWLS can be readily adapted to measure the reaction products of enzymatic reactions. For example, an optical waveguide coated with urease was used to detect the presence of urea via the production of a colored pH indicator [108]. The absorption peak of the indicator was matched to the light in coupled into the waveguide in order to gain sensitivity through the anomalous dispersion of the indicator. One surprising result was that the activity of the adsorbed enzyme adsorbed to the waveguide was about 40,000 times less than that of the enzyme in solution. While complete inactivation of a proportion of the adsorbed enzymes could account for this result, there is other evidence for significant delay in adsorbed enzyme action—the photocycle of adsorbed bacteriorhodopsin runs about 1000 times slower than the protein in its native state, a result that cannot be accounted for by assuming total inactivation of part of the population. The aqueous environment in the vicinity of a surface is known to affect protein structure [109,110], and dynamic properties are, in general, especially susceptible to even small structural changes.

### 7.5. Measurements with Living Cells

These have already been exhaustively discussed in Section 6. It seems safe to say that OWLS, including the RWG, has considerably transformed the ability to quantitatively measure the evolution of cell characteristics, going far beyond the conventional microscopic imaging approach [111,112]. This is very useful for assessing biocompatibility (Section 7.6). Previously, biosensors could be deployed for assessing the adsorption of biopolymers from biofluids, which is part of the problem of biocompatibility, but now the same technology can be used for directly assessing the response of living cells to the material under examination. At the same time, arrays of microwells equipped with individual RWGs have enabled massively parallel screening experiments to be conveniently carried out.

### 7.6. Biocompatibility Sensing

The number of biomedical implants is continually growing. At present approximately 10% of the population of the USA already has an implanted medical device, and it is estimated the number of devices implanted annually is approaching 100 million worldwide; these numbers are increasing because of growing prosperity, which encompasses lengthening life expectancy without concomitant maintenance of full bodily functionality, deficiencies in which can be compensated by implanted devices. They include orthopedic replacement and reinforcement, cardiovascular valves, pacemakers and stents, dental implants, various kinds of neurological implants, surgical mesh, catheters and devices to continuously deliver drugs, such as insulin pumps [113]. However brilliant the technical innovation embodied in the primary functionality of the device, however, it will fail upon implantation if it is not biocompatible, which means essentially ‘tolerant of life’. The implanted medical device industry still relies heavily on in vivo animal testing [114], with some in vitro testing of hemocompatibility being undertaken.

At the same time there is a vast growth in the variety and sophistication of biomedical materials [115,116], and nanotechnology is enabling the fully rational design of biomaterials. There is, therefore, an urgent and rapidly growing need for effective techniques for assessing biocompatibility. The versatility of OWLS makes it highly appropriate to meet this need. The measurement of single protein adsorption kinetics under carefully controlled conditions, for which OWLS is very well suited, is often a good starting point. OWLS has also been applied to the measurement of proteins adsorbed from serum [117]. The complexity of the process was already pointed out by Vroman in pioneering work [118], but OWLS enables this complexity to be dissected and understood. A further strength of OWLS is ability to separate, in a single in situ measurement, the adsorption of protein molecules from changes in surface-resident cell shape [111].

### 7.7. Electrochemical OWLS

The development of transparent but electrically conductive materials such as tin-doped indium oxide (ITO) opens the possibility of combining electrical and optical phenomena. The optical attenuation of ITO, notwithstanding its visual transparency, makes it unsuitable for waveguiding, but functional grating couplers can be fabricated by evaporating a thin layer onto a standard pyrolyzed sol–gel waveguide. Application of an electric field perpendicular to the plane of the waveguide, with an aqueous medium covering it, results in voltage-dependent coupling [119]. This effect could be used, for example, to tune the effective refractive index of a lightmode so close to cut-off that a very small quantity of captured analyte would suffice to extinguish propagation [94], opening the way to ultra-high sensitivity.

Actual applications of electrochemical OWLS have gone in a number of different directions. The application of an electric field has been mainly used to steer the adsorption of proteins and other polymers to the waveguide surface [120,121,122] and to control the stress applied to bacteria, the survival of which on the waveguide surface was being monitored [123]. More recent developments have been reviewed [124,125].

## 8. Conclusions

Above all thanks to its ability to simultaneously measure multiple independent parameters characterizing the solid–liquid or solid–gas interface (which, in biosensing, typically comprises a capture layer), OWLS manifests itself as probably the most powerful general-purpose biosensing technology. Other advantages are the great variety of substrates and wavelengths that can be used, enabling a measuring setup to be well tuned to the problem under investigation.

The trade-off of this power is the greater complexity of the measuring setup, which has hitherto made OWLS unsuitable for competing with compact, mass-market, medical biosensors such as the lateral flow immunoassay. On the other hand the great detail with which interfacial biosensing processes can be investigated makes OWLS a pre-eminent choice for developing other technologies (such as the lateral flow immunoassay), which can be simulated in an OWLS setup. This capability is particularly useful for optimizing the design of capture layers, mass fabrication procedures, and parameter choices for the sensor in use. Hence OWLS can rightly be designated a ‘biosensor of biosensors’.

Regarding the often-discussed question of sensitivity and lower limit of analyte detection, apart from the intrinsic capabilities of the techniques that one is comparing (cf. Table 2), practical matters such as long-term stability (drift), referencing, capture layer design and fabrication all contribute to the overall capabilities. The full power of biosensing is not achieved by a single measurement but by the evolution of binding and the structure of bound analyte which may, in its most sophisticated manifestation, encompass living cells. Very often the precision with which the biophysicochemical conditions in the immediate environs of the biosensing event can be controlled constitute the factor determining sensitivity.

## 9. Future Developments

The immense power and flexibility of OWLS means that, despite having been launched about 30 years ago, there are still spacious domains of unexploited applications, even just within the biosensing field. Some developments that can be anticipated are:

- Simultaneous measurement of parallel fluidic channels on the same grating for referencing purposes;
- Multiwavelength OWLS and combining incoupling angle scanning (as in classical OWLS) with narrow-band wavelength scanning (as in the RWG interpretation of OWLS);
- Near cut-off operation combined with an applied voltage to poise the waveguide very close to cut-off [94,119];
- Exploitation of fluctuation analysis for enhancing biosensing, as has been demonstrated for resistive chemical sensors [126];
- Exploitation of fluctuation analysis for characterizing living cells, analogously to the measurement of electrical resistive fluctuations [127,128].

Doubtless many more will occur to readers.

## Figures and Tables

**Table 1:** Comparison of different techniques in terms of the dependences of waveguide parameters on the obtainable quantities.

		Optical Invariants	
Technique	Noninvariant	1st Order ^a^	2nd Order ^a^
OWLS	${\tilde{\gamma }}_{e}$	$J_{1}$	$J_{22},J_{23}$
Ellipsometry		$J_{1}$	$J_{23}$
SAR		$J_{1}$	$J_{22},J_{23}$

^a^ in $d_{A}/\lambda$.

**Table 3:** Binding of promastigate surface protease (PSP) to a lipid bilayer (POPC) and binding of antibodies to the PSP [70] H-PSP (‘hydrophilic’) lacks a membrane anchor.

Substrate	Analyte	Result/pmol cm^−2^	Interpretation
Lipid bilayer	PSP	1.7	Lipid bilayer covered to the jamming limit
1.7 pmol PSP on lipid bilayer	Anti-PSP	1.7	Specific binding of the antibody to its antigen
Ditto	Nonspecific antibody	0.05	Absence of nonspecific binding to a receptor-saturated transducer
Lipid bilayer	Ditto	0.5	Considerable nonspecific binding on receptor-free lipid bilayer
Lipid bilayer	H-PSP	0.36	Weak binding of PSP lacking a membrane anchor

## Data Availability

No new data were created in this review, hence data sharing is not applicable.
